# Solid phase crystallization of amorphous silicon at the two-dimensional limit†

**DOI:** 10.1039/d2na00546h

**Published:** 2022-12-06

**Authors:** Daya S. Dhungana, Eleonora Bonaventura, Christian Martella, Carlo Grazianetti, Alessandro Molle

**Affiliations:** a CNR-IMM Unit of Agrate Brianza Agrate Brianza I-20864 Italy cgrazianetti@mdm.imm.cnr.it alessandro.molle@mdm.imm.cnr.it; b Dipartimento di Scienza dei Materiali, Università degli Studi di Milano-Bicocca via Cozzi 55 20125 Milano Italy

## Abstract

The epitaxy of silicene-on-Ag(111) renewed considerable interest in silicon (Si) when scaled down to the two-dimensional (2D) limit. This remains one of the most explored growth cases in the emerging 2D material fashion beyond graphene. However, out of a strict silicene framework, other allotropic forms of Si still deserve attention owing to technological purposes. Here, we present 2D Solid Phase Crystallization (SPC) of Si starting from an amorphous-Si on Ag(111) in atomic coverage to gain a crystalline-Si layer by post-growth annealing below 450 °C, namely Complementary Metal Oxide Semiconductor (CMOS) Back-End-of-Line (BEOL) thermal budget limit. Moreover, considering the benefit of the 2D-SPC scheme, we managed to write crystalline-Si pixels on the amorphous-Si matrix. Our *in situ* and *ex situ* analyses show that an in-plane interface or a lateral heterojunction between amorphous and crystalline-Si is formed. This amorphous-to-crystalline phase transformation suggests that 2D silicon may stem from an epitaxially grown layer and thermal self-organization/assembling.

## Abstract 2

We present a novelty in the synthesis of 2D ultrathin silicon on Ag(111) and silicene-on-Ag(111) by molecular beam epitaxy.

## Graphical abstract

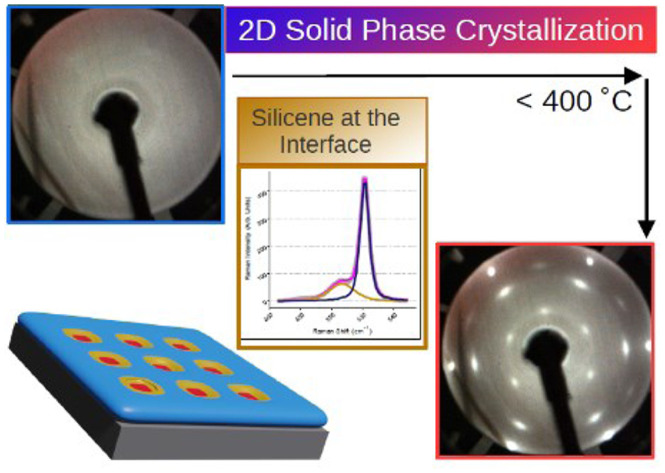

## Introduction

Miniaturization of 2D silicon (Si) has been instrumental in pushing complementary metal oxide semiconductor (CMOS)-based nanoelectronics towards its limit. This scaling is significantly reflected in the dimensional reduction of the active body channel in devices such as transistors.^1,2^ The two major allotropes of ultrathin 2D Si are diamond-like and graphene-like. The diamond-like Si is the conventionally exploited form even inside the more advanced device architectures (*e.g.* Fin Field-Effect Transistor (FinFET)^3^ and multi-bridged nanosheet transistors^4^) and of the frontier mainstream. However, as the name implies, the graphene-like Si,^5^ namely silicene, refers to the newly discovered atomically thin form retaining the hexagonal symmetry. Silicene in a short time has rekindled Si-based applications, such as nanoelectronics,^6^ optics,^7^ and energy.^8^ Similarly, ‘Xene’ refers to the family of graphene-like materials beyond graphene.^9–12^

Owing to the Ag(111) substrate commensurability with the monolayer silicene, silicene-on-Ag(111)^13–19^ configuration is one of the most explored growth cases in Xene synthesis. This growth can be articulated into three categories based on the growth temperature.^13–19^ First is the low temperature growth (∼100 °C) in which the deposition results in amorphous-Si on Ag(111). Second is the medium temperature regime (∼200 °C) in which monolayer silicene appears in mixed crystalline phases (named using the superstructure notation): 4 × 4, √13 × √13 R13.9° type-I and II in addition to some other minor reconstructions. The third one is the high-temperature growth (∼300 °C) in which 2√3 × 2√3 R30° mixes with 4 × 4. Higher temperatures result in 2D layer degradation and eventually in desorption. In the last two cases, the appearance of √3 × √3 R30° (with respect to the silicene lattice) structures marks the starting of the second layer and from the second layer onwards √3 × √3 R30° phase grows on top of the underneath mixed phase.

Because amorphous-Si at the 2D level has no structural affinity with silicene, it can be considered the background to trigger an amorphous-to-crystalline phase transformation. To date, it remains untouched in the silicene growth context; it has previously been demonstrated that it is possible to achieve amorphous-to-crystalline phase transformation. This is usually referred to as Solid Phase Crystallization (SPC)^20–25^ and is one of the preferred ways to synthesize crystalline-Si that are used in solar cell^26,27^ by heating the thick amorphous-Si at an elevated temperature (500–600 °C). Therefore, it could be interesting to achieve SPC on even thinner amorphous-Si at the 2D level with a thickness of few atomic layers.

In this regard, we present the 2D-SPC of amorphous-Si deposited on Ag(111) at low temperatures by Molecular Beam Epitaxy (MBE). First, we show the phase transformation in a large-scale area throughout the sample of ∼10 × 10 mm^2^. Based on *in situ* and *ex situ* monitoring during each process stage, we show that this evolution can be accomplished by heating the sample at 300 °C, namely at a much lower temperature than that used for bulk silicon (∼500–600 °C) owing to the thickness in a few atomic layers. In addition, we show that the 2D-SPC can be effectively translated/reproduced locally using an electron beam (e-beam) irradiation by selectively writing crystalline-Si areas, hereafter termed ‘Si pixels’, on the 2D amorphous-Si layer. A comprehensive analysis of this in-plane heterojunction, in which even silicene fingerprints were hinted, is discussed in detail.

## Experimental

The growth and *in situ* low-energy electron diffraction (LEED)/Auger characterization were carried out in a LAB10 MBE system by ScientaOmicron. A complete sample synthesis comprises three stages: (i) preparation, (ii) growth and (iii) encapsulation. First, the commercially available Ag(111) were cut (∼10 × 10 mm^2^) and loaded inside the MBE system. These Ag(111) wafers were prepared with cycles of 20 min sputtering with Ar^+^ (1 kV/10 mA), followed by successive annealing at 550 °C for 20 minutes, which was first verified using sharp Ag:1 × 1 LEED spots and then contaminants free with Auger. Following the preparation, Si was deposited at 100 °C from an Si sublimator (SUSI by Dr Eberl MBE Komponenten GmbH). In agreement with previously reported silicene growth calibration,^17,18^ the calibration of the flux was set and assigned as a monolayer (ML) Si, which gives a 20–25% of Auger attenuation of the main Auger Ag peak if growth was carried out at 225 °C. The growth rate was fixed at 0.03 ML per second. After observing the amorphous structure of the grown Si layer with LEED, two methodologies were implemented to achieve a 2D-SPC. In the first methodology, aiming at large area crystallization, the amorphous-Si samples were heated for 15 minutes at 300 °C on the same MBE manipulator. In the second methodology, aiming for selective area/spot crystallization, crystalline ‘Si pixels’ were written with high energy electron beams (2.5 keV; 0.5 μm spot size) using the LEED-Auger filament source. The last step is the deposition of an amorphous Al_2_O_3_ capping layer, thereby allowing *ex situ* investigation. The details of this encapsulation can be found elsewhere.^28^

Once the samples are unloaded from the MBE system, *ex situ* micro-Raman characterization was performed using a Renishaw InVia spectrometer. The spectrometer is equipped with a 514 nm (2.41 eV) solid-state laser and a 50× (0.75 numerical aperture) objective. Similarly, the incident laser power was kept below 1 mW to avoid sample damage.

## Results

**Fig. 1:**
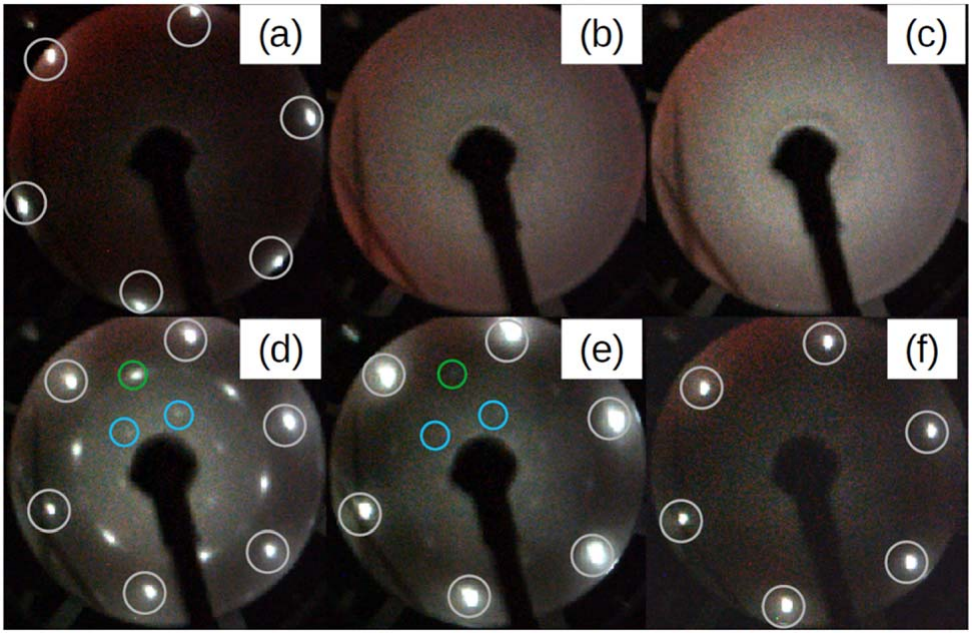
Series of LEED patterns acquired at various stages. (a) Ag(111) surface after preparation and before Si deposition. (b) After depositing a nominal coverage of 2 monolayers of Si at 100 °C. (c) After annealing the surface in (b) for 15 minutes at 250 °C. (d–f) After 15 minutes of annealing of the surface in (b) at 300, 350, and 400 °C, respectively. White circles represent the Ag:1 × 1 spots. Green and blue are respectively Si integer and √3 × √3 spots with respect to integer order Si spots inside the green circle. All LEED images are taken at incident energy *E*_i_ = 50 eV.

Fig. 1 depicts a series of LEED images probing the diffraction pattern evolution as a function of the annealing temperature (*T*_a_). First, sharp diffraction spots from the (1 × 1) Ag(111) surface are clearly visible post to the substrate preparation (see Fig. 1(a)). After that, there are no LEED patterns in Fig. 1(b) that was taken after the deposition of 2 MLs Si at 100 °C, thus confirming the total coverage of Ag(111) substrate with an amorphous structure. The 2 MLs sample was chosen to ensure robustness against many rounds of annealing in this particular case. The amorphous phase continued to appear even when *T*_a_ was increased to 250 °C, as shown in Fig. 1(c). However, when the heating was carried out at *T*_a_ = 300 °C, the surface is no longer amorphous and additional LEED spots along with those from the (1 × 1) Ag(111) surface appear. This brings evidence of a 2D-SPC at ∼300 °C. Similarly, when the same sample was further heated to *T*_a_ = 350 °C, the LEED spot intensity starts to decrease (Fig. 1(e)), and it finally vanishes at *T*_a_ = 400 °C (Fig. 1(f)) where the LEED pattern resembles the one in Fig. 1(a), thereby suggesting a complete Si desorption. The LEED patterns in Fig. 1(d) and (e) illustrate features typical of the √3 × √3 superstructure. As aforementioned, in the context of silicene-on-Ag(111), this suggests the complete wetting of the Ag(111) surface with the first silicene monolayer and at least the second silicene layer in progress (we refer to this configuration as Si:√3 × √3 in the following). Fig. S1 of ESI1† compares the Auger spectra acquired after preparation, which further confirms the Si deficiency on the surface when heated at 400 °C. Additionally, we notice that the thermal budget required to achieve phase transformation is significantly reduced to ∼300 °C with respect to that required for thicker amorphous-Si (500–600 °C). A possible explanation for this gain in the thermal budget should be the dimension reduction at the start with amorphous-Si, which is ultra-thin.

**Fig. 2:**
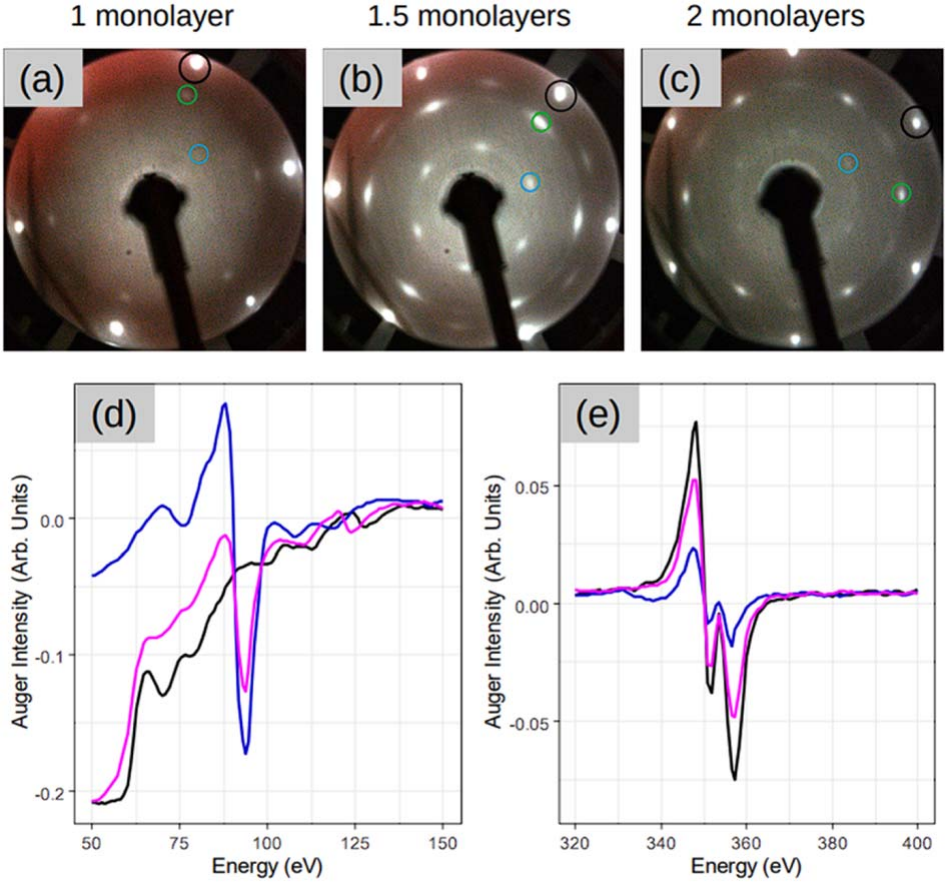
(a–c) LEED patterns acquired after depositing 1, 1.5, and 2 monolayer coverage respectively on Ag(111) at 100 °C, followed by annealing at 300 °C for 15 minutes. Represented once, black circle is Ag:1 × 1 spot, green is Si integer order spot and blue is Si:√3 × √3 spots with respect to integer order Si spot inside the green circle. LEED images (a–c) are taken at incident energy *E*_i_ = 54 eV. (d and e) The Auger spectra acquired at various stages on sample in (c) focusing on the Si peak at 92 eV and Ag peak at 354 eV respectively in (d) and (e). The three color spectra are represented as follows: black is a post to preparation on bare Ag(111) surface, blue is before annealing once amorphous-Si is deposited and pink is after annealing at 300 °C for 15 minutes.

Fig. 2 demonstrates three sample coverages of 1, 1.5, and 2 MLs of amorphous-Si achieved by tuning the growth duration at a given flux and temperature. This sample series was considered because we encountered Si desorption with nominal coverage <1 ML, as shown in ESI2.† After annealing at 300 °C, in the reported LEED patterns, Si:√3 × √3 phase dominates in all the cases, and the patterns differ by 30° rotation. This observation may suggest that the amount of material plays a special role during the phase transformation in determining the majority of rotational domains at the equivalent thermal energy provided. For instance, at low coverage, *i.e.* 1 ML (Fig. 2(a)), a crystalline pattern barely managed to appear. However, when the coverage was increased to 1.5 and 2 MLs (Fig. 2(b) and (c)), the LEED patterns are sharp, therefore suggesting competition between two processes: desorption and crystallization.

For the sample with 1.5 MLs, the Auger spectra of Si and Ag are plotted in Fig. 2(d) and (e), respectively, at different stages of the process. In particular, the black lines are the spectra acquired after the initial substrate preparation, the blue lines are after Si deposition at 100 °C, and the pink lines are the spectra after SPC. It is noteworthy that the Auger spectra after Si deposition at 100 °C (blue lines), combined with the absence of a LEED pattern (Fig. 1(b)), suggest the formation of a relatively thicker Si (few MLs) in amorphous form. This is made clear by the main Ag peak that attenuates significantly (∼70% with respect to the black spectrum Ag peak) and the sharp appearance of the main Si peak at 92 eV. A possible explanation for the observed thick Si deposition is that at low growth temperatures, the adatom diffusion length decreases significantly. In addition, the re-evaporation is reduced significantly because adatoms are landing on a “cold” surface. However, after crystallization (pink spectrum), the Ag peak attenuation readjusts back to ∼35%, suggesting that the Ag(111) surface should have been wet completely with the first and second layers towards the completion, thereby fully confirming a very thin Si layer that is in an atomic layer thickness regime. This suggests that at 300 °C the Si adatoms should have undergone thermally induced self-organization. The consequence of this thermal diffusion kinetics on the surface is the solid phase transformation of amorphous-Si into a crystalline-Si sheet as confirmed by the appearance of the LEED pattern (Fig. 2(b)).

The LEED patterns in Fig. 1 and 2 show no signs of 4 × 4 and √13 × √13 R13.9° silicene phases, respectively. As elaborated in the Introduction, these are the dominant silicene phases when silicene is grown directly on Ag(111). In the new growth approach that we reported here, the LEED patterns in Fig. 1 and 2 favour the √3 × √3 Si(111) phases. This connotes the growth process decisive for the final crystalline Si phases observed. Thus, these two processes follow distinct growth kinetics. In the first case, Si adatoms have sufficient diffusion length because of the moderate growth temperature that ensures crystal growth from the beginning.^29^ In the second case, because the adatoms encounter a ‘colder’ surface, the diffusion is limited that even forbids the formation of a crystalline layer, and the deposition occurs in the form of a bulk amorphous layer. Thus, the first step in this growth approach is to create a direct contact between a crystalline substrate and an amorphous layer. This is confirmed by Fig. 1(a) and (b), where the amorphous Si film (Fig. 1(b)) shadows the Ag:1 × 1 spots that were previously visible before Si deposition in Fig. 1(a). As detailed in the literature by Olson and Roth,^21^ the crystalline substrate now recasts as a template for the crystallization process of an unordered amorphous film to an ordered crystalline layer upon adequate thermal annealing. In a layer-by-layer fashion, the phase transformation of the crystalline layers occurs. This suggests that for our thicker samples the crystallization process should have undergone from the first layer and then the following layers. The reappearance of Ag(111) spots along with the √3 × √3 Si spots after 2D-SPC considerably indicates the same.

**Fig. 3:**
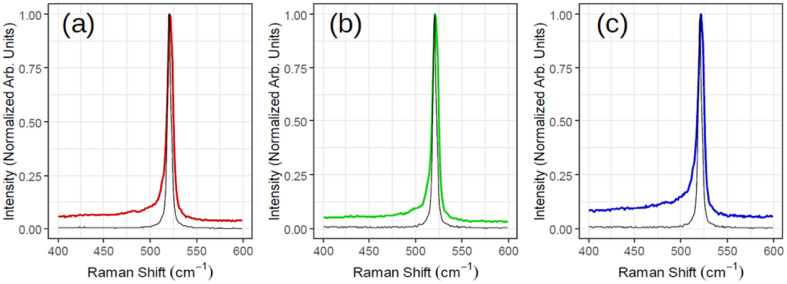
Raman spectra acquired for three samples corresponding to Fig. 2 with (a) red (1 monolayer), (b) green (1.5 monolayers) and (c) blue (2 monolayers). The black spectrum of each subplot is the reference Si for that sample.

Fig. 3 illustrates *ex situ* Raman analysis carried out on the same sample series (1, 1.5 and 2 MLs thick) to have a complementary insight into the 2D-SPC. Before unloading from the MBE reactor, the samples were capped with non-reactive amorphous Al_2_O_3_ to avoid silicon oxidation. The Raman spectra (Fig. 3(a)–(c)) reveal a nearly constant Raman shift and the characteristic Si peak placed at ∼521–522 cm^−1^. However, the most crucial observation is that the Si peak is neither asymmetrical nor that there are any convincing signs of additional components located at lower wave vectors (400–520 cm^−1^). Consequently, none of the Raman spectra can validate typical silicene-on-Ag(111) fingerprints.^30,31^ In addition, the observed Si:√3 × √3 structure may be reconciled with an induced diffusion of Ag atoms from the substrate as reported on monolayer silicene grown at ∼200 °C when heated to 300 °C by Solonenko *et al.*^32^ In that case, the *in situ* Raman spectroscopy indicated the transformation from silicene to diamond-like Si-nanocrystals. Therefore, the crystallized Si reflects a diamond-like form in a 2D regime of ultra-low thickness.

**Fig. 4:**
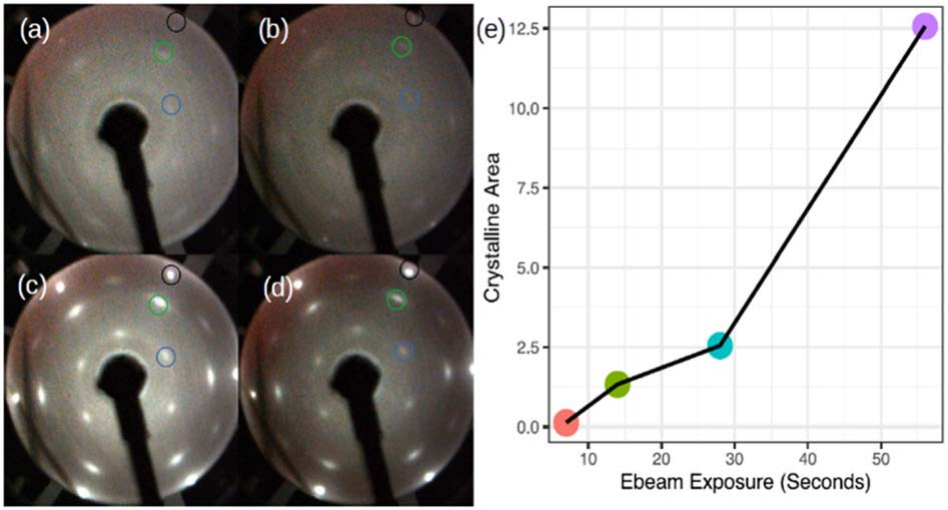
LEED images showing the spot crystallization obtained after exposing the amorphous Si-on-Ag(111) surface with a high-energy electron beam (2.5 keV). The exposure duration is set as follows: (a) 7 seconds, (b) 14 seconds, (c) 28 seconds, and (d) 56 seconds. (e) Is the plot of the nominal crystalline area (mm^2^) with respect to the e-beam exposure duration (a–d). The nominal Si coverage deposited on Ag(111) was 1.5 monolayers. Black circle Ag:1 × 1 spots, (represented once) green: integer order Si spots and blue: Si:√3 × √3 spots with respect to the integer order Si spots inside the green circle. LEED images (a–d) are taken at an incident energy of *E*_i_ = 54 eV.

Now, we turn our attention to spot crystallization where we aim to validate the 2D-SPC using a local e-beam exposure instead of the integral heating of the sample. Fig. 4(a)–(d) shows the LEED patterns acquired immediately after the e-beam exposure on a particular region of the amorphous-Si surface with 1.5 MLs thickness using the same LEED/Auger e-beam source. The beam energy is set constant at 2.5 keV while the four levels of exposure duration, 7, 14, 28, and 56 seconds, were applied on different sites of the sample. First, even with a very short e-beam exposure, *i.e.* 7 seconds in Fig. 4(a), pale diffraction features appear in the LEED pattern from the diffuse pattern of the amorphous-Si state. Second, both the Ag:1 × 1 (black circle in Fig. 4(b)–(d) and Si:√3 × √3 R30° (blue and green circle in Fig. 4(b)–(d)) LEED spots become progressively brighter with an increase in the exposure time. At 28 seconds (Fig. 4(c)), the pattern evolves similar to the one obtained in large area crystallization (see Fig. 1(d) for comparison), thereby displaying intense and well-defined diffraction spots. This suggests that an equivalent thermal budget to that provided by the annealing at 300 °C should have been generated by the local e-beam exposure, thus resulting in a localized crystallization, namely a ‘spot 2D-SPC’. Fig. S3 of ESI3† depicts the LEED images taken along one spot in two in-plane orthogonal directions (lateral and longitudinal) of the sample surface, which shows the evolution of LEED patterns. From the LEED images in ESI3,† it is clear that the LEED patterns start to diminish once we move away from the spot centre; at one point, no LEED patterns can be observed at all. Therefore, we can conclude that there is a co-existence of amorphous and crystalline-Si in the same sample surface. In other words, a lateral heterostructure is made by placing amorphous-Si and crystalline-Si on the same substrate, thus achieving crystalline-Si pixels that are embedded in an amorphous-Si matrix by e-beam writing.

To facilitate the *ex situ* Raman analysis of the Si pixel, a ‘Region of Interest (ROI)’ was created in the amorphous sample through several e-beam exposures (5–10 times; 2.5 keV for 56 s) (ESI4†). Consequently, the manipulator was moved slightly in the *xy* plane (assigning *z* orthogonal to the surface or parallel to the e-beam). Therefore, the crystalline surface is the fusion of several pixels on the amorphous-Si matrix. The reason for opting for this approach is that we were unable to locate even the matrix of pixels on the sample in the *ex situ* environment. This confirms that the spot area is much smaller when compared with the nominal one made with manipulator reading. In the following, we detail the *ex situ* Raman analysis made on this ROI that is inside the amorphous-Si.

**Fig. 5:**
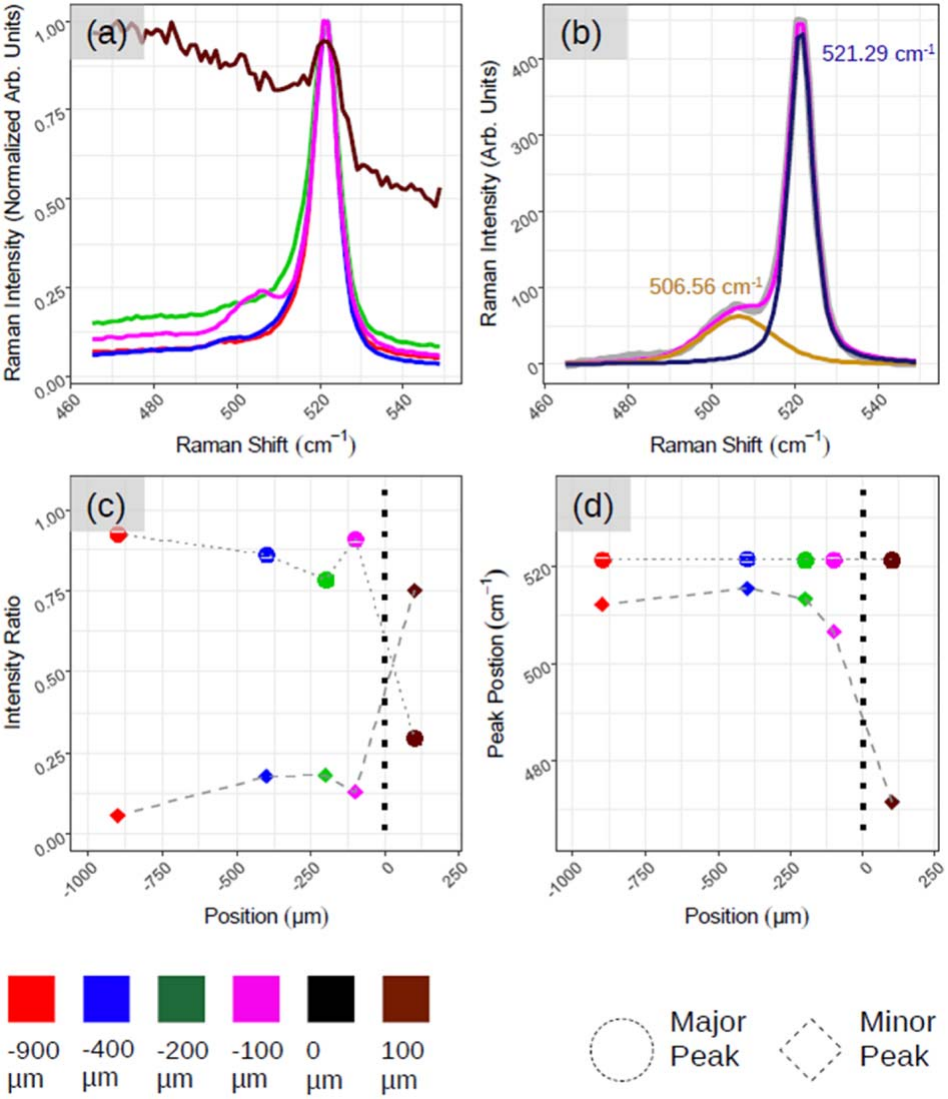
Demonstrates the Raman analysis of the interface between amorphous-Si and crystalline-Si. (a) As acquired Raman spectra as a function of position. (b) Fitted Raman spectrum corresponding to pink spectrum in (a) where the gray curve is raw data, pink is the fit result, dark-blue and orange are two used Lorentzian–Gaussian components. (c and d) Quantification of spectra in (a) and (b). (c) The intensity of major and minor components with respect to position. (d) The location of major and minor Si-related Raman peaks.

Fig. 5 demonstrates the positional Raman spectroscopy carried out on the interface between the aforementioned ROI and amorphous-Si. Two regions can be observed in Fig. 5(a): one characterized by a sharp Si-related peak in magenta, green, blue and red spectra, and the other region characterized by a broad spectral band, a brown spectrum. The shape profile of this brown spectrum (see ESI5 Fig. S5(a)† for more spectra) agrees with that of phonon modes in amorphous materials in which the interatomic bond length distribution is highly dispersed. From these two observations and for convenience, we arbitrarily assign this interface co-ordinate (*D*) as the origin, where *D* > 0 is an amorphous region and *D* < 0 belongs to the crystalline region. However, what is most striking is the emergence of an additional peak component that is highly pronounced at the interface region (Fig. 5(a), magenta spectrum at *D* = −100 μm) as shown with two components of Lorentzian–Gaussian fitting (magenta curve in Fig. 5(b)) after the background subtraction. This gives the accurate position of the Raman peaks, 521.29 cm^−1^ and 506.56 cm^−1^, for principle (dark blue curve in Fig. 5(b)) and secondary peak (orange curve in Fig. 5(b)), respectively. ESI5 Fig. S5(b)† illustrates additional spectra close to this region, which further substantiate this secondary Si peak that makes the spectrum asymmetrical in ∼100–200 μm range at the junction. To quantitatively assess the relative weight between the principal and secondary peaks (referred to as major and minor peaks in the following, respectively), we deconvolved the Raman spectra with two fitting components after background subtraction.

In Fig. 5(c), we plot, as a function of the spatial position, the relative weight of each peak calculated as the ratio, *I*_peak_/*I*_tot_, where *I*_peak_ is the intensity of the (major, minor) peak and *I*_tot_ is the sum of the peak intensities derived by the fitting procedure. The solid circle and diamond represent major and minor peaks, respectively. Fig. 5(d) shows the centre of the two peaks where we can confirm the major Si peak at 521–522 cm^−1^ and minor one at ∼490–500 cm^−1^ in all the cases. The emergence of asymmetry with the pronounced side peak is further confirmed in Fig. 5(c). For instance, moving from the red to magenta spectrum, we can notice that the spectral separation between the major and minor peaks (represented respectively with dots and bricks) is reduced.

These findings converge towards a ‘silicene-in-junction’ in between the crystalline-Si and amorphous-Si that should be in a few atomic layers in terms of thickness. First, the black spectrum (ESI5 Fig. S5(a)†) along with no LEED patterns (Fig. 1(b)) and Auger spectra as depicted in Fig. 2(d) and (e) confirm a thick amorphous region (∼6 MLs).^15^ Second, the red Raman spectrum (Fig. 5) with Si:√3 × √3 LEED patterns (Fig. 4) suggests a diamond-like crystalline-Si as observed before with large area crystallization. Furthermore, this red spectrum shares identical Raman spectra features: (i) lacking asymmetry, (ii) almost no secondary component and (iii) main Si peak located at 521–522 cm^−1^ that further consolidate this.

The major reason for these signatures should be the thermal energy gradient, which the ebeam spot creates around its centre, once we move away from the pixel centre. At one point (corresponding to −100 μm or the pink curve in Fig. 5), the thermal energy should have been sufficiently low enough to prevent excessive Si desorption and forbid the complete transformation from a graphene-like to diamond-like phase. This furthermore resulted in the junction thickness between the diamond-like crystalline-Si and amorphous Si (ESI5 Fig. S5(c)†). Most importantly, the curve is asymmetrical with a side hump, which agrees with the fingerprint of the multilayer silicene on Ag(111).^33^

As we already noticed, spot crystallization *via* 2D-SPC is possible well below <450 °C, thus opening the avenue for low-thermal budget applications of locally crystallized nanoscale Si. Patterning Si spots (pixels) within an amorphous-Si 2D matrix with low-temperature processing is compliant with the CMOS process flow, provided that silicene is eliminated from the Ag below.^34^ This full CMOS compatibility^35^ makes spot crystallization suitable for embedding silicene transistors in a pre-patterned CMOS platform with a thermally compliant process scheme. We believe that this can be an additional tool for the route towards Gate-All-Around (GAA)^36–38^ silicene FETs.^39^

## Conclusions

In conclusion, we investigated SPC on amorphous-Si on Ag(111). We demonstrated, with *in situ* LEED/AES and *ex situ* Raman spectroscopy, that SPC at the 2D level occurs at ∼300 °C, which is well below the CMOS BEOL thermal budget of 450 °C. Similarly, we described a method to achieve SPC by selectively writing ‘Si pixels’ with an e-beam source that can be engineered further by adjusting free parameters. We believe that this new growth approach to writing ‘Si pixels’ at the 2D level can be interesting in future nanomaterial engineering routes by applying more precise (*e.g.* focused e-beam^40^) or different (*e.g.* laser^41^) heating printers.

## Author contributions

DSD conceived the idea, planned the growths and the *in situ* analysis. EB acquired and analyzed the *ex situ* Raman spectroscopy data. DSD wrote the manuscript with inputs from the other authors. All authors read and approved the manuscript. CM, CG and AM coordinated the research tasks. AM supervised the research project.

## Conflicts of interest

There are no conflicts to declare.

## Supplementary Material

NA-005-D2NA00546H-s001
