# Supplementary material for: Solid phase crystallization of amorphous silicon at the two-dimensional limit
Source: Nanoscale Adv. 2022 Dec 6;5(3):668–74. doi: 10.1039/d2na00546h (PMC9890642; doi:10.1039/d2na00546h)
Supplement: NA-005-D2NA00546H-s001 [file NA-005-D2NA00546H-s001.pdf]

Electronic Supplementary Material (ESI) for Nanoscale Advances.
This journal is © The Royal Society of Chemistry 2022

Supplementary Information

Solid Phase Crystallization of Amorphous Silicon at the Two-Dimensional Limit

Daya S. Dhungana[1], Eleonora Bonaventura[1],Christian Martella[1], Carlo Grazianetti[1*], Alessandro Molle[1*]

[1] CNR-IMM Agrate Brianza Unit, via C. Olivetti 2, Agrate Brianza, I-20864, Italy

*Corresponding authors: carlo.grazianetti@mdm.imm.cnr.it, alessandro.molle@mdm.imm.cnr.it

KEYWORDS: Silicene, Solid Phase Crystallization, Molecular Beam Epitaxy, 2D Materials

SI1: Auger Spectra

S12: LEED Patterns below 1 Monolayer

SI3: Methodology for Nominal Thickness

SI4: Region of Interest with Si Pixels Merger

S15: Raman Analysis of Region of Interest

SI1: Auger Spectra of sample after 400 °C Annealing

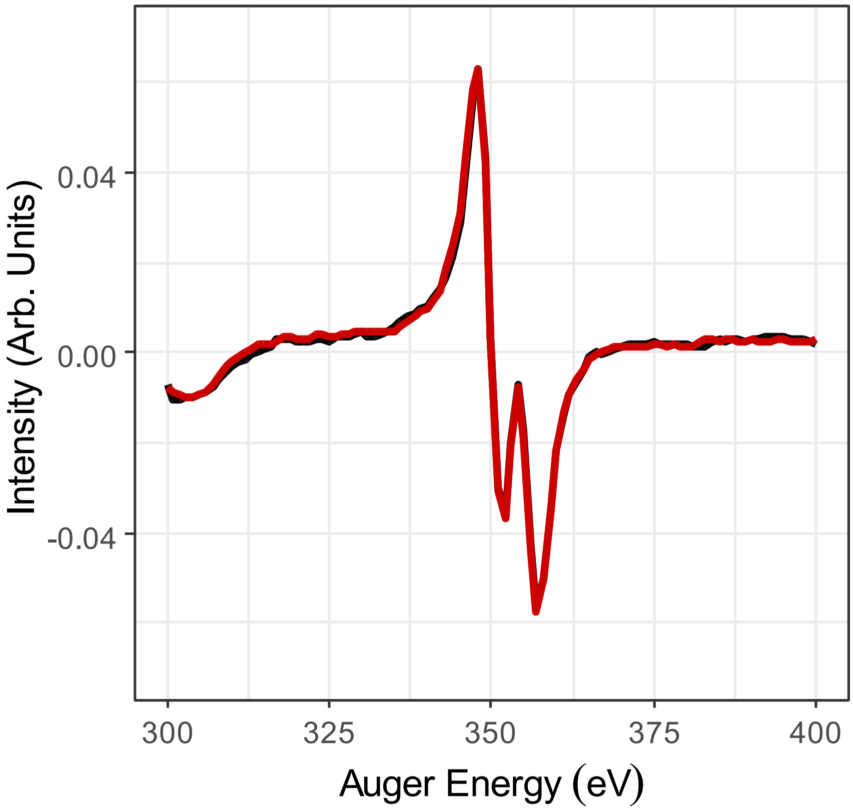

Figure S1: Auger spectra acquired on two different surfaces: (i) black before depositing Si (ref. main text **figure 1a**) and (ii) red after annealing deposited Si at 400 °C (main text **figure 1f**).

SI2: LEED Patterns below 1 Monolayer

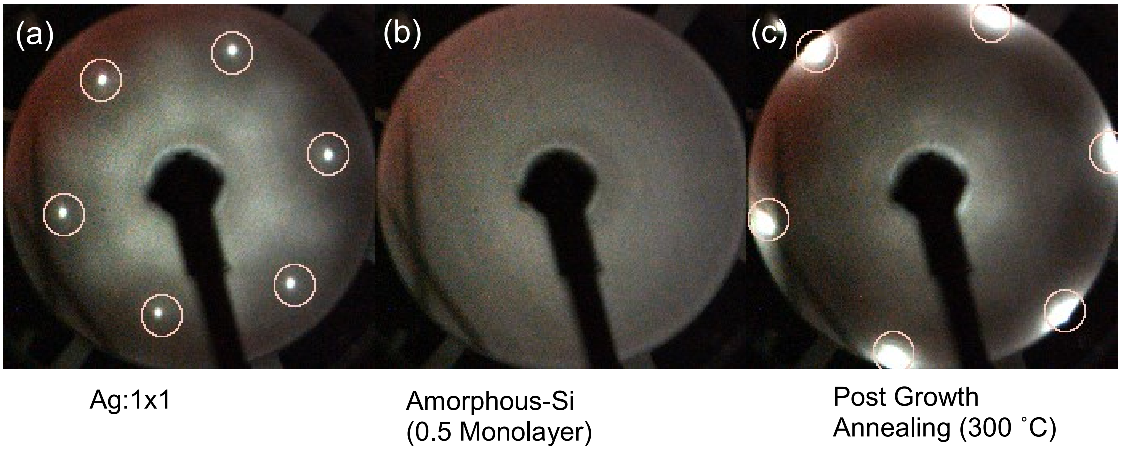

Figure S2: (a) Ag:1x1 surface post to preparation. **(b)** Amorphous-Si after depositing 0.5 monolayer of Si on Ag(111). (c) LEED patterns observed after heating surface in **(b)** at 300 ˚C for 15 minutes. Incident LEED Energies (E_i) for **(a)**, **(b)** and **(c)** respectively are 90, 50 and 50 eV.

SI3: Methodology for Nominal Thickness

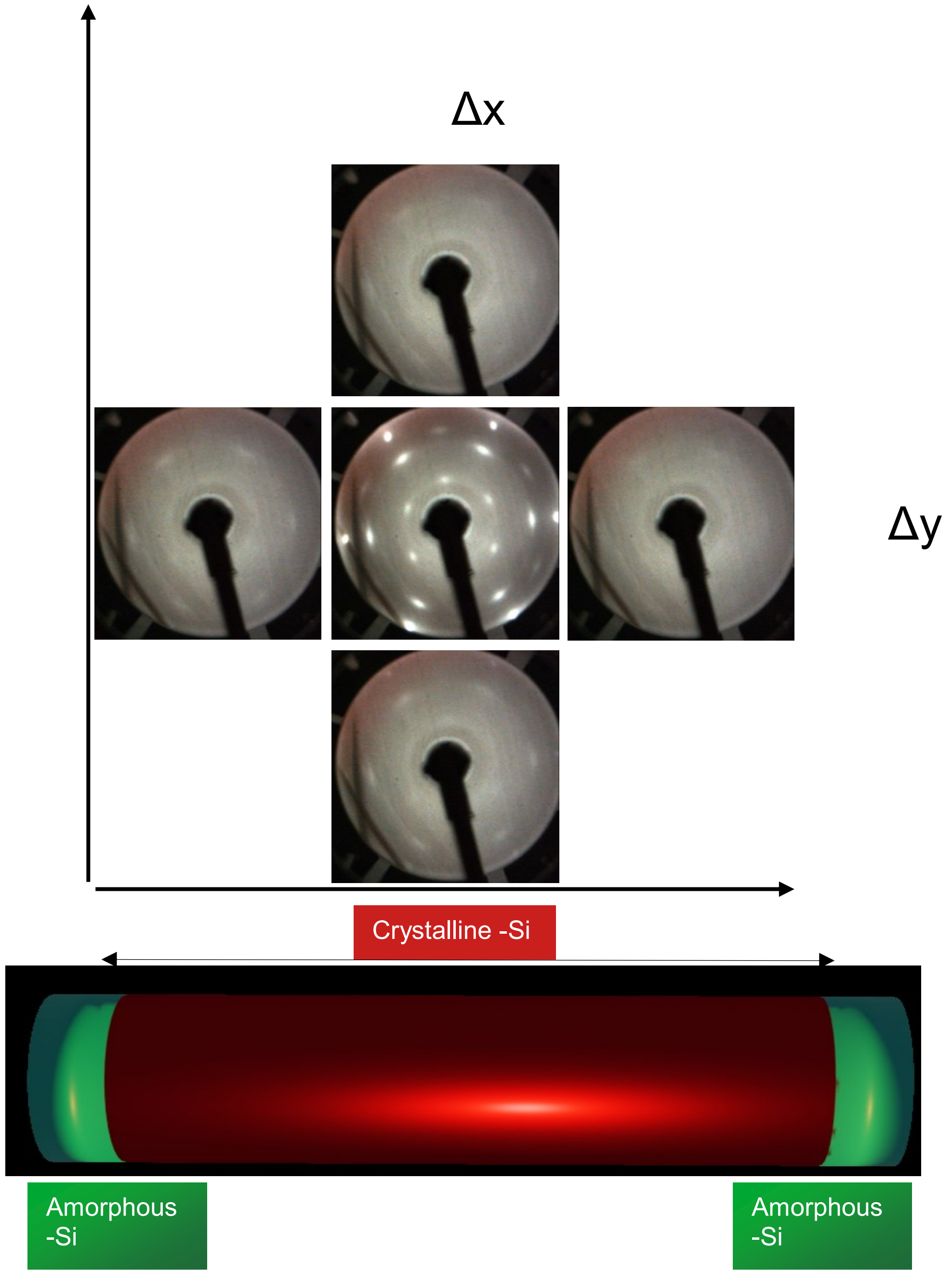

Figure S3: Methodology to determine nominal crystalline length with the help of manipulator. The manipulator is moved along in one axis at a time, either x or y (z is perpendicular to the sample surface) until amorphous region (green) appears at the end. This gives the crystalline length.

SI4: Region of Interest with Si-Pixels Merger

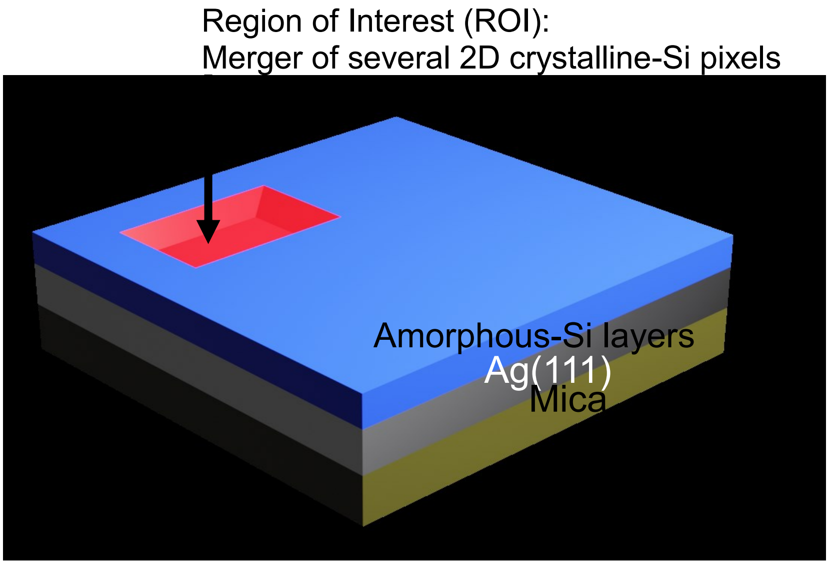

Figure S4: 3D sketch of Region of Interest (ROI) that was created on Amorphous-Si layers

SI5: Raman Analysis of Region of Interest

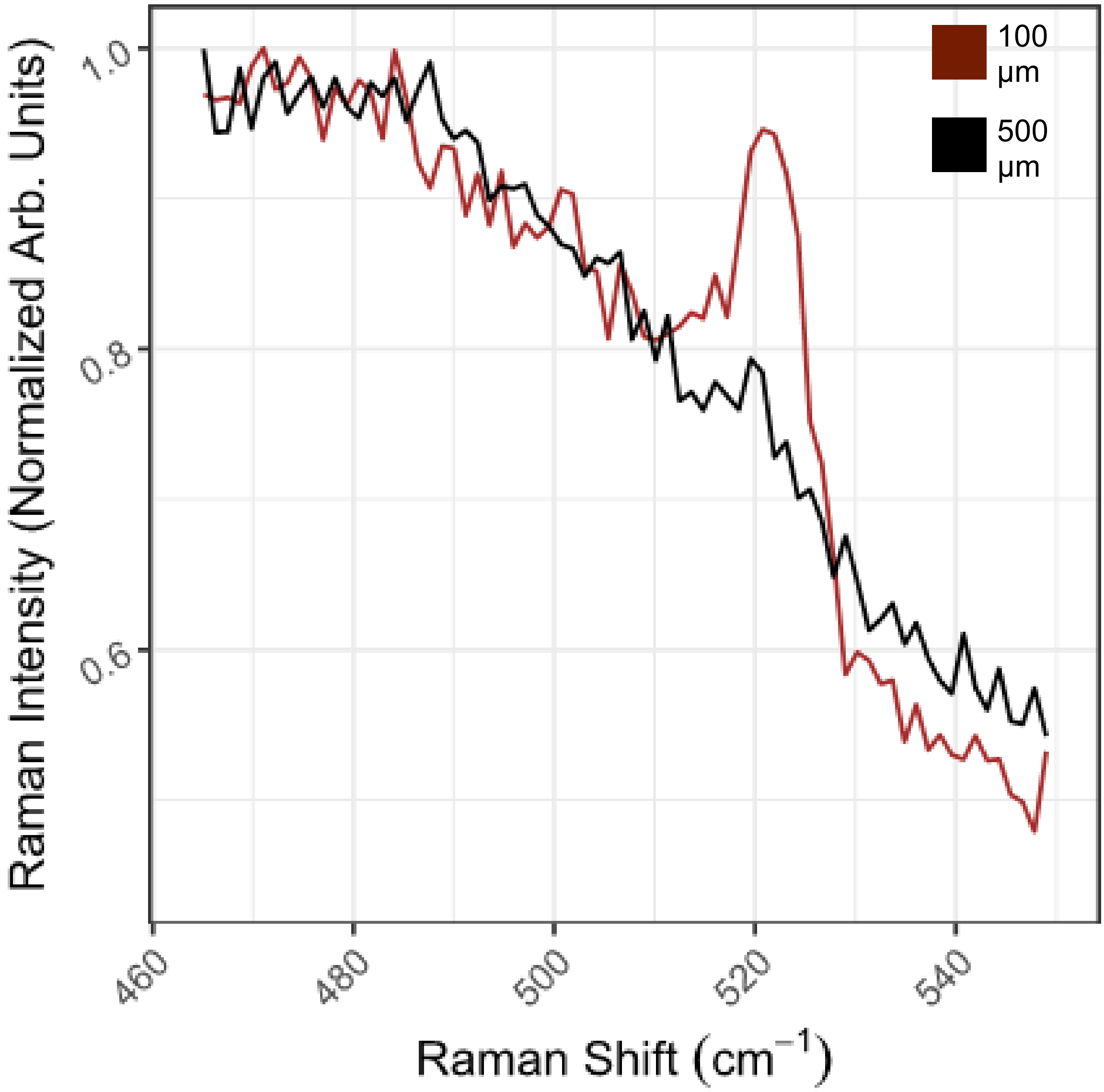

Figure S5a: Raman measurements while moving towards amorphous-Si side from crystalline-Si side.

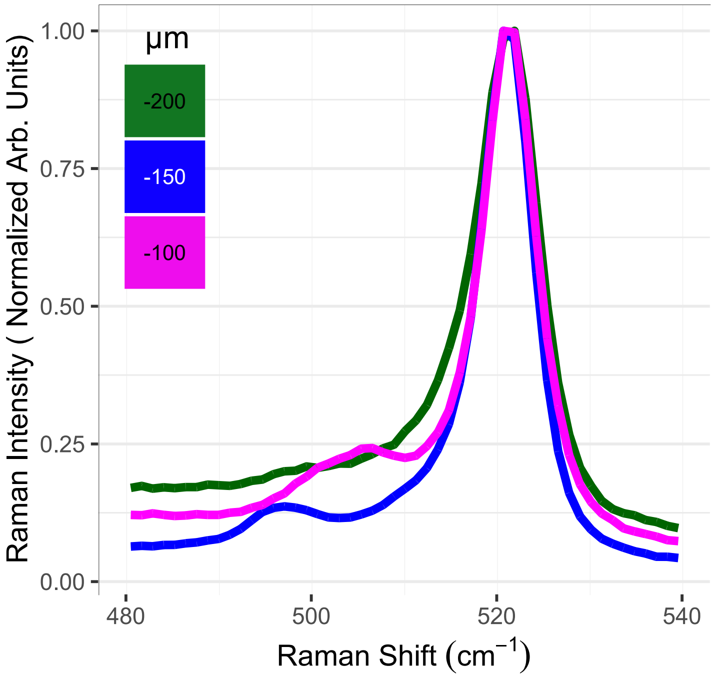

Figure S5b: Raman spectra at different positions between crystalline and amorphous-Si interface at Region of interest (**figure S4**). (D=-50 µm; purple), (D=-100 µm; orange), (D=-150 µm; magenta) and (D=-200 µm; black). Crf: **Figure 5** (main text)

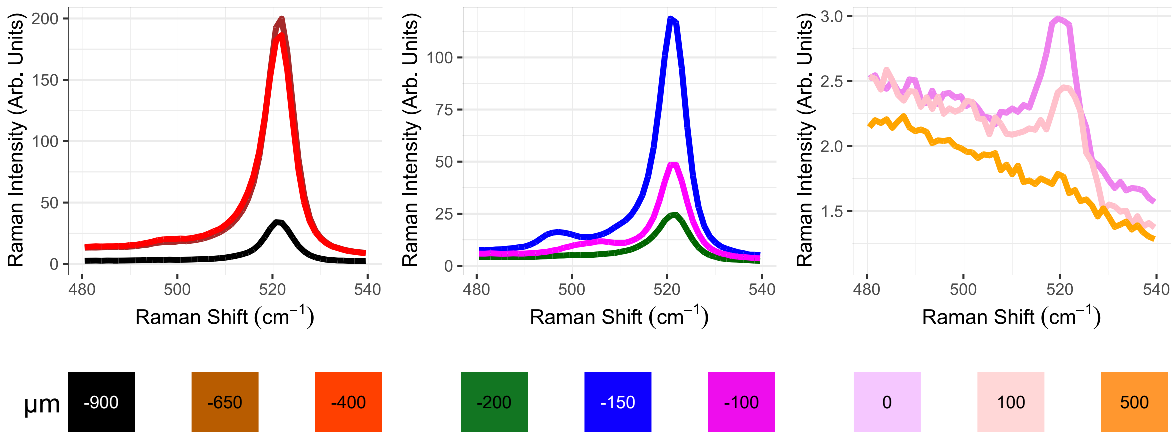

Figure S5c: As acquired Raman spectra along a scan while moving from crystalline (left) to amorphous region (right) at Region of Interest (**figure S4**). Crf. **Figure 5** (main text).
